# Multiple influences on participating in physical activity in older age: Developing a social ecological approach

**DOI:** 10.1111/hex.12608

**Published:** 2017-08-02

**Authors:** Elisabeth R. Boulton, Maria Horne, Chris Todd

**Affiliations:** ^1^ Division of Nursing, Midwifery and Social Work School of Health Sciences Faculty of Biology, Medicine and Health University of Manchester Manchester UK; ^2^ Manchester Academic Health Sciences Centre Manchester UK; ^3^ School of Healthcare Faculty of Medicine and Health University of Leeds Leeds UK

**Keywords:** adherence, older adults, physical activity, social ecological approach, uptake

## Abstract

**Background:**

Evidence of the benefits of engaging in physical activity (PA) is strong, yet the number of older adults meeting the recommended 150 min/wk is low. Policy to increase uptake and adherence has focussed on the health benefits of PA, but may not be the most successful approach.

**Objective:**

This qualitative study sought to ask older adults what the components of a successful intervention to promote PA would be, by asking active older adults what motivated them to be active and asking inactive older adults what might encourage them to change.

**Design, setting and participants:**

Focus groups and semi‐structured interviews were held with 60 community‐dwelling older adults, aged 50‐87 years. Framework analysis was used to analyse the data, and themes congruent with a social ecological model of behaviour change were developed.

**Findings:**

Five themes emerged that influenced PA engagement at multiple levels: individual; interpersonal; perceived environment; community or organizational; and policy. PA engagement was determined by attitude or health status for some participants, but for the majority, PA being enjoyable, sociable, affordable, accessible, flexible and seasonal were more important than the health benefits.

**Discussion and conclusions:**

A social ecological model is presented, highlighting the fact that both motivated and unmotivated older adults need to have a range of appropriately labelled, appealing and accessible activities to choose from when thinking about engaging in PA. Policymakers and practitioners need to ensure that their offers of activity sessions are easy to access and easy to remain involved in.

## 1 INTRODUCTION

The health benefits of physical activity (PA) are well documented with higher levels and greater frequency of PA being associated with reduced risk and improved health in a number of key areas.^1^, ^2^, ^3^, ^4^ Improvements in mental health, well‐being and cognitive function are also associated with regular PA.^5^, ^6^, ^7^, ^8^ Despite these health benefits, PA levels amongst older adults remain below the recommended 150 min/wk.^1^, ^9^ International and national health policies have focused on improving health by providing PA guidance to the older population^10^, ^11^, ^12^ and by highlighting to those working with older adults the need to incorporate increased amounts of PA into everyday life.^1^, ^13^

Contemporary policy developments in the UK have sought to involve people in improving their own health and well‐being, to maximize the opportunities for better health outcomes.^14^, ^15^ Participative approaches have been used in the management of long‐term conditions, nutrition and addiction,^16^, ^17^ and there are suggestions that this could be fruitful in other areas. A literature review examining the involvement of older adults in the design, delivery, implementation and promotion of interventions to increase PA found only ten studies in which older adults played an active and prominent role.^18^ Of these, only four studies demonstrated that older adults were involved in designing and promoting an intervention, based on factors that would appeal to their age group.^19^, ^20^, ^21^, ^22^ In the wider literature, there are a number of examples of older adults developing individual action plans and coping plans within interventions,^23^ but not in designing the structure of the overarching interventions, which are delivered within particular environmental, organizational and policy structures.^24^

Social‐ecological approaches to public health interventions provide a framework for understanding the importance of the dynamic interrelations between a person and their environment, and the context within which they exist, recognizing the complexity of human situations.^25^ Whilst recognizing the importance of psychosocial factors on behavioural change, such as knowledge, attitudes, beliefs, self‐efficacy and social support,^26^, ^27^, ^28^, ^29^, ^30^ Social Ecological Models (SEMs) also include consideration of influences at organizational, environmental and policy levels.^24^, ^25^, ^31^

The aims of this qualitative study were to (i) explore the views and experiences of older adults in relation to successful PA interventions and (ii) to develop recommendations with older adults for a population level PA intervention to promote uptake and adherence. The research question was as follows: “What do older adults think the essential characteristics and preconditions are for the delivery of a successful intervention to promote physical activity amongst their age group?” The study follows a pragmatic approach to consider influences on PA engagement at multiple levels,^32^, ^33^, ^34^, ^35^ leading to the development of a SEM for promoting PA amongst older adults.

## 2 METHODS

### 2.1 Participants

Community‐dwelling older adults, aged 50 years and older, were recruited through non‐government organizations and community groups in West Yorkshire, UK, being purposively and snowball sampled to recruit participants with different experiences of participation or non‐participation in PA. Due to limited resources, people who did not speak English, or who had other significant communication difficulties requiring a translator, were initially excluded. An amendment was approved to include two participants who spoke Urdu. Recruitment continued until no novel data were produced.^36^, ^37^ Participants provided informed consent prior to taking part in the study and agreed to audio recording of responses. The study received ethical approval from the University of Manchester's Committee on the Ethics of Research on Human Beings.

### 2.2 Data collection

Prior to the recruitment of participants, a public involvement consultation exercise was undertaken with 12 older adults to formulate and clarify the questions that would be asked in focus groups and interviews.^38^ Topics included participants' understanding of PA; the PA undertaken by participants; their reasons for uptake, adherence and termination of PA; their views on group and individual PA, including environmental and social factors; and their views on the promotion of PA amongst their age group. The majority of participants recruited opted to take part in focus groups between October 2011 and April 2012. Over the same period, semi‐structured interviews were conducted with those who preferred to meet 1:1 or were unable to attend focus groups. Data collection took place in community buildings and participants' homes.

### 2.3 Data analysis

Given the relevance of this study to the national policy framework regarding promotion of PA, prevention and healthier communities, the framework approach to data analysis was used.^39^ This approach facilitates systematic qualitative analysis, summarizing and classifying data within a thematic framework.^40^ The focus group and interview recordings were transcribed verbatim and initially coded using NVivo9^41^ by the first author, before meeting with the other authors. Classification began following the first three data collection events and continued throughout the data collection period. Through discussion, all three authors developed a thematic framework containing key themes and subthemes. This framework was systematically applied to all of the data, being modified and refined through five phases of review and analysis. All authors met to discuss and develop the framework at regular intervals, classifying and synthesizing data, reviewing themes and questioning linkages in the data. The research findings were presented to 20 study participants at two community events, for credibility.^42^

## 3 RESULTS

Eleven focus groups and 12 interviews were conducted. Participants were aged between 50 and 87 years, with the majority being female. The gender, age, ethnicity and level of PA of the 60 participants recruited are reported in Table 1. Participants were described as physically active if they met the current recommendations.^3^

**Table 1:** Participant characteristics (n=60)

Characteristic	Category	n
Gender	Female	46
	Male	14
Age (years)	50‐64	27
	65‐74	23
	75‐84	9
	85+	1
Highest education	No further education	25
	Some further education	35
Ethnicity	White British	57
	South Asian	2
	Black British	1
Level of physical activity	<150 min/wk	34
	>150 min/wk	26

**Figure 1:**
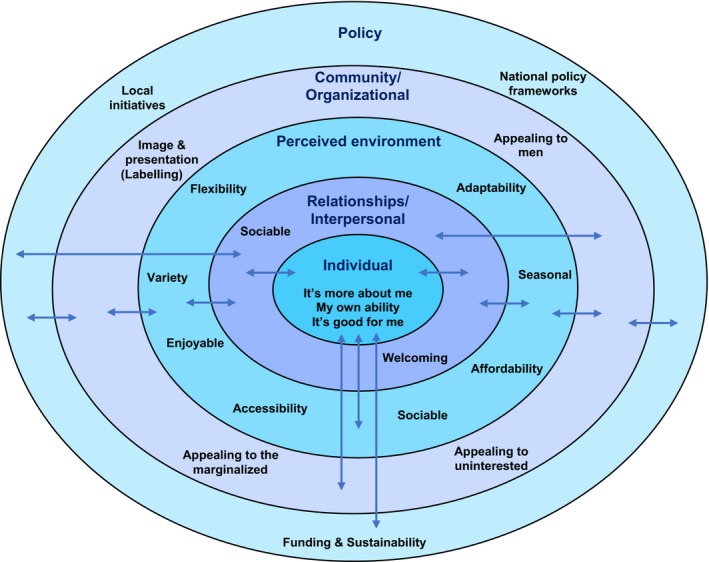
A social ecological model for promoting physical activity amongst older adults

Five themes emerged that influenced PA engagement at multiple levels of the SEM: individual/intrapersonal; relationships/interpersonal; perceived environment; community/organizational; and policy levels. The SEM developed from the findings is presented in Figure 1. Exemplar quotes are included in the main text, with additional supporting quotes presented in Table 2. Quotes from participants meeting the recommendations for PA are indicated by the word “Active” preceding their gender and age.

**Table 2:** Supporting quotes from participants by SEM level

SEM level and quote number	Quotes
*Intrapersonal or individual level*	
Quote 1	“Sometimes you feel you'd like to go to something to learn something, rather than it just be a physical exercise.” (Active Male, 66)
Quote 2	“This is the thing about me dog, when it's windy or rainy, you've got to go anyway. I'm walking because I'm going somewhere. I walk because I walk me dog, I wouldn't just go for a walk.” (Active Female, 56)
Quote 3	“I want to do these things, but my body won't let me. I want to overcome it and I find I physically can't.” (Female, 66)
Quote 4	“If you don't get off your backside and do something then, you know, you are going to end up permanently on your arse, you know what I mean?” (Active Male, 66)
Quote 5	“Table tennis has been a saviour for me really. As a focus for my physical activity, but also just to occupy me mentally.” (Active Male, 65)
*Interpersonal or relationships level*	
Quote 6	“It's that social, community, that we enjoy as much as anything else. Meeting everybody and socialisation more than the actual gardening.” (Active Male, 66)
Quote 7	“It's the best thing I ever did, coming down here. I joined people who were like meself you see.” (Female, 80)
*Perceived environment level*	
Quote 8	“I'm getting a bit fed up now. Once you've learnt it, we're just going over the same things.” (Active Female, 56)
Quote 9	“One [leader] is a fast walker and one isn't; one at the front and one at the back and you've got little off‐shoots… I think they have a fantastic group and there's some in their 80s!” (Active Female, 56)
Quote 10	“Pay per class, then if you're strapped for money, you don't have to pay it. You've not got to find a big lump sum to start with.” (Female, 76)
Quote 11	“I'm very grateful for the bus pass, I wouldn't be going out at all without this.” (Active Female, 63)
Quote 12	“I love curling, I absolutely love it! Oh it's a laugh, it's a hoot! It's really good, it really is good!” (Active Female, 64)
Quote 13	“Ice, snow and ice, I'm frightened to death of falling. The snow yes, but it's the ice. These two winters that we've had, it's just been a nightmare.” (Active Female, 69)
*Community or organizational level*	
Quote 14	“They don't like being organised. Probably because they've been organised all their working life and when they get to the stage of retiring, they don't want to.” (Female, 54)
*Policy level*	
Quote 15	“I bought a [curling] set myself. By the time we've found a grant, we might have missed all these men who've come along. It cost me £300.” (Active Female, 63)

### 3.1 Individual/intrapersonal level

Here, participants talked about their own ability, motivation and belief in the health benefits of PA. The influence of participants' personalities and lifestyles was described and reflected upon.

#### 3.1.1 It's more about me

There was a range of engagement in PA amongst participants. From very little activity (walking to the shops, travelling to a social activity) to PA dominating their week (active in groups, or on their own, on every day of the week). The most commonly reported driver for PA within the very active subgroup was that they had always been active; they were unable to imagine not being active. “*I can't survive without doing it. That's how intense mine is, it's just a part of me life. I couldn't possibly imagine not doing any of it.”* (Active Female, 66) For some, the desire and ability to learn something new was the motivating factor to engage in activities *(*Table** 2 *, Quote 1)*. For others, their perceived inability to learn something new prevented engagement. Ten participants talked about having a dog and being active every day. Two inactive participants stated that they had stopped walking regularly when their dogs died, despite their best intentions to continue *(Quote 2)*.

#### 3.1.2 My own ability

Feeling fortunate to be fit was expressed in three of the focus groups. “*The way I look at it, I'm lucky to be able to get about the best way I can. So I'm just glad about that.”* (Female, 78) In contrast, many participants spoke of their frustration at ill health and poor mobility restricting them. For some, this frustration was increased because they had previously been active *(Quote 3)*.

#### 3.1.3 It's good for me

Whilst the health benefits of PA were not spoken about by many participants, for some they were a major driver. The responses included recovery from illness or surgery; improving health; avoiding ill health and preventing decline; losing and maintaining weight; and achieving mental well‐being. “*I come to make my health better. I make the time to come.”* (Female, 61) *(Quote 4)* Mental stimulation, keeping their brains active, was described by participants who engaged in some of the more tactical physical activities like bowling, curling and table tennis. *“You use your brain too; you watch and work out. You're on the move all the time.”* (Female, 64) A small number of participants talked about the problems they had with depression and the positive relationship between PA and their mental health *(Quote 5)*.

### 3.2 Relationships/intrapersonal level

Here, participants described the social influences and benefits that they experienced.

#### 3.2.1 Sociable

The social element of PA was rated highly by many. Meeting new people, forming friendships and socializing with like‐minded people were often more attractive than the activity itself *(Quote 6)*. Companionship and company were particularly important factors for participants who lived alone *(Quote 7)*. Several talked about forcing themselves to go out to combat loneliness. “*I live on me own and I get fed up on me own. The main reason for going out is to meet people.”* (Female, 66) For some walking into a room full of strangers was the greatest barrier to engaging in PA. A friendly atmosphere and a designated person to welcome new people into a group or activity session were crucial for first‐time attendance. “*I do think that the meet and greet has a lot to do with it. If someone comes and welcomes you in, then you feel ‘well yes, I can do this!’ if you're on your own, or new to being on your own.”* (Active Female, 76)

### 3.3 Perceived environment

Here, participants talked about the need for sessions to be easy to access, not just in terms of physical access (on a bus route, with good car parking, no steps), but also through atmosphere and composition.

#### 3.3.1 Flexible, variable and adaptable

Flexibility was discussed in relation to the freedom that people had once they had retired. Signing up for a course requiring regular attendance was not desirable when increased leisure time meant they could spend time in new pursuits and away from home. Some participants talked about travel and family commitments and did not want to tie themselves down to additional regular commitments. “*I've spent a lifetime committing, jumping to the bell. I don't wear a watch now. If sommat better comes along, going to the theatre, having a meal, all rings more bells for me than coming to T'ai Chi.”* (Female, 69) The idea of a “drop‐in” approach to activity sessions and the possibility of having a “taster” session, to try things out, was popular as there was no pressure to commit. Variety, between and within activities, was reported as important. Participants were unlikely to continue to engage in activities that became boring *(Quote 8)*. Adaptable activities were important so that people could go at their own pace within an activity. Instructors needed to ensure that everyone could take part at their own pace, without feeling hurried or inadequate *(Quote 9)*.

#### 3.3.2 Affordable

The majority of participants were retired with pensions of varying amounts. Only five participants were in employment and two were on work‐related benefits. Difficult decisions about how to spend limited income were reported. “*I'd go more often, but it's a bit, money's a bit tight. I find at the moment that I would like to join a lot more things, but they all cost.”* (Active Female, 76) The loss of subsidised and free activity sessions, such as adult education classes and swimming, meant that some participants' opportunities for activities had been cut off. Paying for a set of classes in advance was out of the question for most, who preferred a “pay‐as‐you‐go” approach, which enabled them to budget and adapt *(Quote 10)*.

#### 3.3.3 Accessible

Being able to engage in PA very close to home was important. Access to venues needed to be as easy as possible. “*Easy access. That's what we've liked about this. We're not reliant on t'buses. It's within walking distance for most of us, innt it?”* (Female, 78) They spoke of the free bus pass (given to pensioners in the UK) acting as a facilitator of PA; it encouraged them to go out more *(Quote 11)*. The importance of the local environment was discussed in terms of both quality of venues and the beauty of the local area. Venues needed to be clean and warm, as well as close to home. There did appear to be a relationship between the outdoor environment and the amount of PA undertaken. Of the 15 participants who spoke of the attractiveness of the local area, ten were regular walkers and five were motivated to walk, although not regularly.

#### 3.3.4 Enjoyable

Enjoyment was mentioned most often regarding engagement in PA and was evident in both group and independent activities. Many had difficulty articulating why they engaged in PA beyond the fact that they enjoyed doing so. “*I just enjoy it. I'm aware that it's good for me, but lots of things are good for you that I don't do, so that can't really be the reason!”* (Active Male, 66) When talking about an activity that they enjoyed, participants smiled and often laughed, demonstrating a link between enjoyment, laughter and fun *(Quote 12)*.

#### 3.3.5 Weather, seasons and time

Many reported being less active in the winter, less willing to go out after dark and in bad weather *(Quote 13)*. As the days became longer, participants reported increases in their enthusiasm and PA. Evening activities, such as gardening, walking and taking grandchildren to the park, became possible. Participants who were reluctant to attend afternoon sessions, because they finished after dark, reported being more willing to attend in the summer months. “*Morning I think and before it gets dark, although in summer I love early evening. Once it's light in the evening I might go out.”* (Active Female, 64)

### 3.4 Community/organizational level

Here, participants reported various challenges for those encouraging older adults to engage in PA.

#### 3.4.1 Appealing to men

According to participants attending group PA sessions, women dominated membership; there was a perception that perhaps this put men off from attending. “*Unless they feel, you know, a bit female dominated, you know, ‘cause there's only females there and if there's only a couple of men…”* (Active Female, 56) Some women spoke of their husbands' dislike of having regular commitments after decades of employment, stating that there was an unwillingness to be organized in their leisure time *(Quote 14)*. Where men were engaging in organized activity sessions, it appeared to be as a result of an interest in a particular activity, as opposed to a desire to join any group‐based session. The men who were members of the table tennis club spoke of their lifelong interest in the sport, supporting this view. Other PA engaged in by men included T'ai Chi and dancing with their wives.

#### 3.4.2 Appealing to all ages

There was some frustration amongst participants about the labelling of activities for “over 50s.” There was a perception that the age range was too great. Some younger older adults did not identify themselves in the same age group as those in their 70s. The “over 50s” term was described as exclusive, preventing engagement from many who did not identify themselves by their age. “*So once you start putting ages, you're kind of ruling some people out who'd probably try it, you know.”* (Active Female, 64) Many people over 50 are still in work, and the “over 50s” classes were often run during the day. One participant was most emphatic that they were “retired people's classes,” rather than for those over 50.

#### 3.4.3 Appealing to the uninterested

In the discussions about how to motivate the uninterested to engage, there were more questions and recounts of failed attempts than answers and suggestions. Using a variety of methods and resources to promote activities was suggested, with word of mouth being thought to be the most effective tool. “*You need it more than one way as well, don't you? A personal invitation and perhaps something to give them to remind them.”* (Active Female, 64) Thinking about the target audience, and using language that people can identify with, was particularly important for the South Asian Ladies' group “Gup Shup” (Chit Chat), as was conducting the activity sessions within their own locality.

### 3.5 Policy level

A small number of participants talked about affordability in relation to the sustainability of the groups and sessions on offer. A minimum number of attendees were required to cover room hire and leader costs. In some cases, tutors and were donating time and equipment *(Quote 15)*, but room hire costs remained. There was frustration that funding and practical assistance were not offered by the local government and health services, because it was thought that the cost benefits of older adults being more physically active and engaged in their communities were obvious. “*…provision of equipment, materials, space, is not cheap and you'll never be able to do it on the cheap, you know. It's gonna need financial backing.”* (Active Male, 67)

## 4 DISCUSSION

This article addresses the applied public health issue of how to encourage greater levels of PA amongst older adults. To help us achieve practical goals, we have framed this article within a social ecological approach.^24^ We recognize that we could approach this issue from a variety of theoretical perspectives and that each may highlight different factors. However, SEMs have been used successfully to promote PA because individuals, with their own unique motivations, engage in activity that takes place in particular settings and contexts.^30^ SEMs permit inclusion of a broad range of variables, from different domains, and recognize the importance of the dynamic, multidirectional influences between people and their physical, social and political environments.^25^ The interaction of personal, social, environmental, organizational and political variables on health is of particular relevance to policymaking and public health implementation.

The multilevel influences on engagement in PA uncovered by this research are presented in Figure 1 as a SEM for promoting PA amongst older adults. The figure illustrates five levels of influence on PA engagement, placing the individual at the centre and showing that there are multiple factors influencing behaviour. Factors at each level have dynamic, multilevel influences, indicated by the arrows in the SEM.

At the individual level, factors included wanting to learn, being frustrated by poor health or feeling that being physically active was an essential part of identity. Existing habits of exercise have been shown to act as an important motivator to engage in physical activities in older age, as has the establishment of routines for activity.^43^, ^44^ Knowledge of the physical and psychological health benefits of being active can act as an incentive to be physically active, whilst poor health can act as a barrier. This confirms the existing research on promoting physical activity amongst older adults.^29^, ^45^, ^46^, ^47^ The motivation to learn something new leading to uptake of PA, the importance of mental stimulation provided though tactical activities, and avoiding or averting depression through being physically active, appear to be new findings. There is increasing evidence that dog ownership leads to higher levels of PA.^48^ Dog owning participants in this study were physically active on a daily basis, regardless of poor weather or reluctance to go out. The specific influence of having a dog on walking behaviour is evidenced by the fact that participants stopped walking regularly once dog ownership had come to an end, despite their good intentions. A sense of responsibility towards the dog, and the adverse consequences that would occur through a failure to exercise the pet, may override any lack of motivation to walk alone. There is a dynamic relationship between the owner and the dog, which results in regular walking activity. This leads us on to the next level of the SEM.

At the interpersonal level, the social element of engaging in activities was found to be an important motivator to adhere to PA. Relationships were built and maintained through attending groups and activities with others. Opportunities for social interaction with new and existing friends, which encourages engagement in physical activities, have been found in previous studies,^49^, ^50^, ^51^ but the importance of ensuring that activity sessions are welcoming to new members has been less well documented.^52^ This study has highlighted the importance of ensuring that there is a key person nominated to welcome new people to activity sessions, in order to make their first experience a positive one, maximizing the chance of return. The relationships experienced in this level of the SEM contribute to the creation of the perceived environment, which is the next level in the model.

Sallis et al.'s^31^ level of the “perceived environment” is adopted here due to its recognition of the importance of the physical and social characteristics of activities and environments that influence engagement in PA: What makes the activities accessible and appealing? At this level are the categories of flexibility, variety and adaptability; affordability; accessibility; enjoyment; weather, seasons and time. Participants' experiences of these factors influence how they feel and act and thus influence the relationships that they have at the interpersonal level. There is a dynamic, multidirectional relationship between the SEM levels.^25^

The importance of affordable activities has a strong evidence base, to which this study contributes.^49^, ^50^, ^53^, ^54^ Within this study, this has been linked to safe and easy access to activities through public transport, facilitated by the free bus pass for those of State Pension age.^51^ This study confirms the importance of ensuring that PA is enjoyable, as very few people stated that they were active for the health benefits alone.^50^, ^52^, ^53^, ^54^ The outdoor environment has been shown to encourage people to be more active in their local areas,^29^, ^55^ but this is mitigated by the influence of poor weather and dark nights on engagement in PA and attendance at activity sessions.^43^, ^46^, ^47^, ^51^, ^56^, ^57^ This study has found that engagement in PA is seasonal, with activity levels increasing in the warmer months. Some PA interventions are season‐specific, such as outdoor bowling, and alternatives may need to be offered to maintain PA levels throughout the year. There should be variety within activity sessions, to ensure that participants remain interested and engaged.^44^ These are considerations for those organizing PA, leading us into the next level.

At the community, or organizational level of the SEM are findings regarding image and presentation. Activity sessions should be labelled appropriately, in order for people to identify with the activity on offer. Using the “over 50s” label may not be the best way to promote PA amongst this age group, as few people identified as “over 50.”^58^ The age range from 50 years and upwards is too large to be meaningful to every person within that range. Use of the label “over 50” has not been widely reported as a barrier to engagement in community‐based activities for older adults and this warrants further consideration. The action research study following on from this qualitative study considered this issue in more depth, as the researchers sought to find a way of appealing to local community members over the age of 50.

Consideration should also be given as to how to appeal to different subgroups of the older adult population, to men and the “uninterested.” This study has found that there have been difficulties in engaging men in organized PA sessions, which confirms findings of previous research and have led to the development of older men only activities.^59^, ^60^ Within this study, there were greater numbers of female members in organized activity groups. Participants suggested this might be due to a dislike of organized activities, or of spending time in female‐dominated groups. In the UK population, there are fewer older men than older women^61^ which will have some part to play in the gender balance of the PA sessions. After the age of 75 years, there are twice as many women than men living in the locality where this study was conducted.^61^ However, up to the age of 75 years, the numbers are similar.^61^ Other studies have shown that Muslim women are deterred from attending mixed‐gender activities due to their own or family concerns over modesty and gendered norms.^62^ Therefore, to ensure participation of BME groups, facilities congruent with their practices and beliefs need to be developed.^30^, ^62^ A systematic review of interventions to promote PA amongst people with severe mental illness showed that lack of support was a major socio‐economic barrier to engagement.^63^ Organizers and promoters of PA interventions need, then, to ensure that appropriate support is available to facilitate engagement. The challenge of appealing to those regarded as “uninterested” should also be addressed when considering image and presentation. This study confirms the difficulties of attracting older adults who most need to increase their PA levels^64^ and highlights the need for action to be taken at policy levels to address this issue.

At a policy level, much can be done to support the adoption of PA. However, the national policy frameworks and initiatives discussed in the introduction appeared to have had little impact on the participants in this study. None of the participants mentioned the influence of national guidance as a driver for engaging in PA behaviour. That guidance appeared to be undermined by local policy decisions to end subsidies for free swimming and reduced cost adult education, which had a direct impact on the self‐reported PA. Local decisions, which directly affected local people, appear to have had a greater impact on PA engagement than national campaigns to encourage healthy behaviour, although those local initiatives are often driven by national agendas, such as budget cuts, or the need to reduce hospital admissions.^65^ Concerns about the sustainability of activity sessions were raised by participants, who wanted to see local government departments provide both financial and practical resources to support activity sessions.

### 4.1 Strengths and limitations

The participants in this study were recruited through existing community groups and at an “activity showcase” event. Snowball sampling led to recruitment of a further 23 participants. Each participant was either physically active, or involved in some kind of social group activity, or both. Reference was made to family members or friends who did not engage in any activity (social or physical), but they were not directly involved in this study. The study was geographically bound in the north of England, but the methods described mean that it could be replicated elsewhere.

The lead author had worked and volunteered in the area where the study was conducted for more than 15 years at the time the study was conducted. Participants could recognize a long‐standing commitment to older adults in the locality, with knowledge of the local situation. This author had prior knowledge of the activity sessions attended by 33 of the 60 participants, yet the same kinds of factors were reported by the remaining 27 participants, for whom no prior knowledge regarding activities existed. This suggests that the author's existing knowledge had not adversely influenced the understanding and analysis of the findings and adds to the evidence of rigour within the study.

Whilst the overrepresentation of women within this study mirrors the gender balance within the community activities taking place, it does not provide a representative sample of the general population over 50 years of age. The dominance of women within the study could lead to a focus on factors that appeal to women. The feedback received from men within this study indicates that they are more motivated by interest in a particular activity than by social interaction. Thus, caution should be applied in focusing on the social element of PA as a solution for all members of the older population.

### 4.2 Recommendations for policy and practice

Policymakers can do much to improve the promotion of PA at multiple levels of the SEM. As few participants were driven to take part in PA to gain physical health benefits, more focus should be given to the social benefits and the fun, laughter and enjoyment that can be obtained. The labels applied to PA promotion should be as open and inclusive as possible. Ease of access should also be considered, in terms of cost, location, a friendly welcome and timings that reflect seasonal changes. In particular, the temptation to remove the free bus pass for people of State Pension age should be resisted. Local government departments and health services should consider the cost benefits of providing resources to support community‐based activity sessions, not least in creating enjoyable environments, because the health benefits of PA are well‐known.

### 4.3 Recommendations for further research

Findings from this study suggest that the “over 50s” label is perceived as exclusive, yet it is common for initiatives to use this label. Further research into older adults' views on the labelling of groups and activities is required, to see whether this is a major barrier to engagement. Further research is also needed into how to appeal to those perceived as marginalized, disengaged and uninterested in PA. Participatory approaches would be most useful here, to understand the difficulties and develop potential solutions from the perspective of those affected.

## 5 CONCLUSION

This study found that, in some cases, there are personal attributes that override the extrinsic factors that policymakers and local organizations can have some influence on. Poor health, and mobility, feeling the need to be active (or not), and being motivated to engage in activity can be the primary driver for PA engagement. Not everyone will respond positively to targeted advertisement of PA. However, through ensuring that activities are flexible and adaptable, making sure that people feel welcome regardless of ability, could help to encourage older adults to try activities that they might otherwise rule out. Ensuring that activities on offer have maximum appeal is a key element of increasing engagement. Interventions that focus on individual‐level predictors of engagement require attractive activities to signpost older adults to. The dynamic relationship between influences at all five levels of a SEM will assist in the development of interventions that take into account optimum conditions for engagement.

The focus of public health interventions to promote PA has largely stressed the health benefits.^1^, ^9^ However, adopting a multilevel approach to promoting engagement in PA, by focussing on the social elements of engaging in activity, together with ensuring that it is easy to take part, both on a practical and emotional level, may do more to encourage older adults to meet the recommendations of 150 minutes of moderate intensity PA over the course of a week.^1^, ^9^

## CONFLICTS OF INTEREST

The authors declare that they have no proprietary, financial, professional or other personal competing interests of any nature or kind.
